# Association of Sodium-Glucose Transport Protein 2 Inhibitor Use for Type 2 Diabetes and Incidence of Gout in Taiwan

**DOI:** 10.1001/jamanetworkopen.2021.35353

**Published:** 2021-11-19

**Authors:** Mu-Chi Chung, Peir-Haur Hung, Po-Jen Hsiao, Laing-You Wu, Chao-Hsiang Chang, Ming-Ju Wu, Jeng-Jer Shieh, Chi-Jung Chung

**Affiliations:** 1 Division of Nephrology, Department of Medicine, Taichung Veterans General Hospital, Taichung, Taiwan; 2 PhD Program in Translational Medicine, National Chung Hsing University, Taichung, Taiwan; 3 Rong Hsing Research Center For Translational Medicine, National Chung Hsing University, Taichung, Taiwan; 4 Department of Biotechnology, Asia University, Taichung, Taiwan; 5 Department of Internal Medicine, Ditmanson Medical Foundation Chiayi Christian Hospital, Chiayi, Taiwan; 6 Department of Applied Life Science and Health, Chia-Nan University of Pharmacy and Science, Tainan, Taiwan; 7 Department of Urology, China Medical University and Hospital, Taichung, Taiwan; 8 Institute of Biomedical Sciences, National Chung Hsing University, Taichung, Taiwan; 9 Department of Education and Research, Taichung Veterans General Hospital, Taichung, Taiwan; 10 Department of Public Health, College of Public Health, China Medical University, Taichung, Taiwan; 11 Department of Medical Research, China Medical University Hospital, Taichung, Taiwan

## Abstract

**Importance:**

The use of sodium-glucose transport protein 2 (SGLT2) inhibitors is currently a standard intervention in patients with type 2 diabetes (T2DM) and exerts favorable pleiotropic effects to consistently lower blood urate levels. However, to date, no association between SGLT2 inhibitor use and the incidence of gout have been established.

**Objective:**

To investigate whether prescribed SGLT2 inhibitors are associated with lower gout incidence in patients with T2DM.

**Design, Setting, and Participants:**

In a cohort study, all patients with incident T2DM in Taiwan National Health Institution databases between May 1, 2016, and December 31, 2018, were retrospectively analyzed. As a comparator, patients using dipeptidyl peptidase 4 (DPP4) inhibitors were included. A total of 47 905 individuals receiving an SGLT2 inhibitor and 183 303 receiving a DPP4 inhibitor were evaluated, along with 47 405 pairs of patients using an SGLT2 inhibitor or DPP4 inhibitor in 1:1 propensity score–matched analyses. Data analysis was conducted from April 1 to June 30, 2021.

**Main Outcomes and Measures:**

A gout diagnosis was based on the* International Classification of Diseases, Ninth Revision, Clinical Modification (ICD-9-CM)* and the *International Statistical Classification of Diseases, Tenth Revision, Clinical Modification (ICD-10-CM).* Multiple Cox proportional hazards regression models were used to calculate hazard ratios (HRs) and 95% CIs.

**Results:**

In total, 231 208 patients with T2DM were included in the population; 113 812 individuals (49.22%) were women, and the mean (SD) age was 61.53 (12.86) years. The overall gout incidence was 20.26 per 1000 patient-years for SGLT2 inhibitor users and 24.30 per 1000 patient-years for DPP4 inhibitor users. When potential risk factors were adjusted in the propensity score–matched population, use of SGLT2 inhibitors was associated with a lower risk of gout (HR, 0.89; 95% CI, 0.82-0.96) compared with DPP4 inhibitors, particularly for patients receiving dapagliflozin (HR, 0.86; 95% CI, 0.78-0.95). A sensitivity analysis, performed when a gout diagnosis was ascertained using the *ICD-9-CM* or *ICD-10-CM* code with gout-related medication, also showed a significantly lower risk for gout incidence of 15% with SGLT2 inhibitors (HR, 0.85; 95% CI, 0.74-0.97). Subgroup analysis indicated that SGLT2 inhibitor benefits in patients with T2DM to achieve a lower gout risk were not different across subgroups.

**Conclusions and Relevance:**

The findings of this study suggest that patients with T2DM who are receiving SGLT2 inhibitors may have a lower risk for gout compared with those receiving DPP4 inhibitors.

## Key Points

**Question:**

Is the use of a sodium-glucose transport protein 2 (SGLT2) inhibitor for patients with type 2 diabetes associated with the incidence of gout?

**Findings:**

In this Taiwan nationwide cohort study, there was a significant 11% risk reduction in the incidence of gout in 47 405 patients with type 2 diabetes who received an SGLT2 inhibitor compared with 47 405 propensity score–matched individuals receiving dipeptidyl peptidase 4 inhibitors, particularly for patients using dapagliflozin. The benefits of SGLT2 inhibitor use in patients with type 2 diabetes for a lower gout risk were not different across subgroups.

**Meaning:**

The findings of this study suggest that use of SGLT2 inhibitors may help to decrease the incidence of gout in patients with type 2 diabetes.

## Teaser

This cohort study examines the use of sodium/glucose cotransporter 2 inhibitor therapy in patients with type 2 diabetes at risk for gout.

## Introduction

Type 2 diabetes (T2DM) is associated with a risk of hyperuricemia due to insulin resistance or hyperinsulinemia, which decreases urinary urate secretion.^1^ Hyperuricemia in turn is associated with a risk of diabetic kidney disease progression and cardiovascular disease.^2^ Gout development is a severe condition in which cardiovascular comorbidities are often frequent.^3^

Sodium-glucose transport protein 2 (SGLT2) inhibitor administration reduces blood glucose levels and is currently the standard intervention for preventing diabetic kidney disease progression and cardiovascular disease in patients with T2DM. Associations between baseline blood urate levels and cardiorenal outcomes and death have also been observed in a large-scale SGLT2 inhibitor trial.^4^ Mediation analyses also showed that reductions in blood urate levels contributed to modest but significant SGLT2 inhibitor effects in lowering cardiovascular death.^5^

In a previous trial,^6^ different SGLT2 inhibitor medications consistently lowered blood urate levels to 0.3-0.9 mg/dL. In general, the kidney accounts for approximately 70% of urate elimination, and SGLT2 inhibitors could facilitate glucose transporter 9 excretion of more urine uric acid in exchange for glucose reuptake by increasing glucose concentrations in glomerular filtrate.^7^ Because hyperuricemia is accepted as the most important risk factor for development of gout,^8^ we speculated whether SGLT2 inhibitor use influences the risk of gout.

To date, no association between SGLT2 inhibitor use and the incidence of gout have been established. In this study, we used Taiwan’s National Health Insurance database to assess the association between SGLT2 inhibitor use and the risk of gout incidence compared with use of dipeptidyl peptidase 4 (DPP4) inhibitors in patients with T2DM. Furthermore, we explored which patient groups appeared to benefit from SGLT2 inhibitor therapy in terms of lowered gout risk.

## Methods

### Database Information

In 1995, a comprehensive database containing medical information on approximately 23 million Taiwanese citizens was constructed via the National Health Insurance program. This program is one of the national health insurance systems in the world with mandatory registration to satisfy all populations with equal rights for medical care. All records, including outpatient care, hospital care, laboratory test results, drug prescriptions, and interventional procedures, are collected for the declaration of medical expenses. Taiwan’s National Health Research Institute permits the release of database information for research purposes. Patient consent is not required to access the National Health Insurance Research Database. This study was approved by the research ethics committee of China Medical University Hospital. All methods were performed in accordance with the relevant guidelines and regulations of China Medical University Hospital. Our study followed the Strengthening the Reporting of Observational Studies in Epidemiology (STROBE) reporting guideline for cohort studies.

### Study Design and Participants

We established a nationwide retrospective cohort study and used deidentified secondary data from the National Health Research Institute database. This step replaced individual identification numbers with unique numbers to protect privacy and facilitate follow-up. All patients with incident T2DM in the National Health Research Institute database were defined using diagnostic codes from the *International Classification of Diseases, Ninth Revision, Clinical Modification* (*ICD-9-CM*) and *International Statistical Classification of Diseases*, *Tenth Revision, Clinical Modification* (*ICD-10-CM*) (eTable in the Supplement) and at least 3 outpatient visits or 1 hospitalization within 1 year. Diagnostic accuracy was previously verified in this database.^9^ We identified 265 458 patients with T2DM who were initially administered SGLT2 inhibitors or DPP4 inhibitors between May 1, 2016 (the time SGLT2 inhibitors were first used in Taiwan), and December 31, 2018. The first date of SGLT2 inhibitor or DPP4 inhibitor use was defined as the index date. All index dates occurred after T2DM was diagnosed.

**Figure 1.**
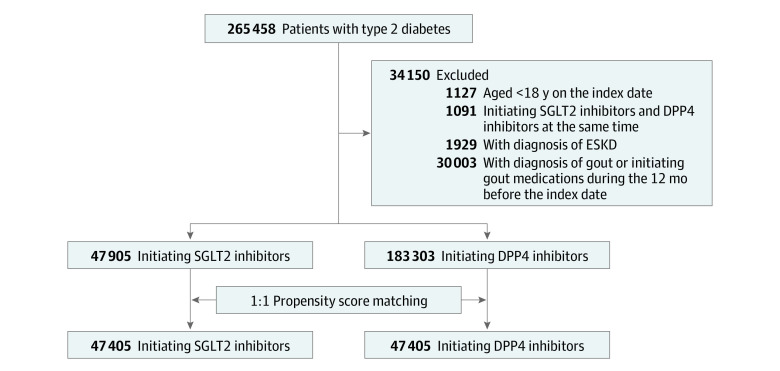
Study Flow and Patient Selection DPP4 indicates dipeptidyl peptidase 4; ESKD, end-stage kidney disease; SGLT2, sodium-glucose transport protein 2.

We excluded patients younger than 18 years, those simultaneously receiving SGLT2 inhibitors and DPP4 inhibitors, and patients undergoing dialysis before the index date. We also excluded patients with a gout diagnosis and those who were using gout-specific medicine (including allopurinol, benzbromarone, colchicine, febuxostat, and probenecid) within 1 year before the index date. We enrolled 47 905 patients with prescriptions for SGLT2 inhibitors and 183 303 patients with prescriptions for DPP4 inhibitors. Furthermore, to avoid confounding effects from baseline comorbidities and medicine use, we performed a 1:1 propensity score–matched analysis of 47 405 pairs of patients with T2DM receiving SGLT2 inhibitors or DPP4 inhibitors based on age, sex, index year, comorbidities, and use of other medicines. Data on race and ethnicity were not collected. A detailed study flow diagram is shown in Figure 1.

### SGLT2 Inhibitor or DPP4 Inhibitor Administration

Medications of interest were SGLT2 inhibitors (including dapagliflozin, empagliflozin, and canagliflozin) and DPP4 inhibitors (including alogliptin, linagliptin, sitagliptin, saxagliptin, and vildagliptin). For SGLT2 inhibitors or DPP4 inhibitors, detailed information on drug type, quantity, dose, dispensing date, and days of drug supply was collected.

### Definition of Gout Outcomes and Other Covariates

The primary outcome was the incidence of gout in the propensity score–matched population. Gout outcomes were established using diagnostic codes from *ICD-9-CM* and *ICD-10-CM* (eTable in the Supplement). Gout is typically diagnosed using criteria from the American College of Rheumatology^10^ and the European Alliance of Associations for Rheumatology. Diagnosis code validity and completeness were both validated in a previous study^11^ in which positive predictive values meeting American College of Rheumatology criteria ranged from 68% to 82% by increased frequency of gout diagnosis. We also followed standard nomenclature for gout.^12^ In addition, we compared the distribution of baseline comorbidities and prescription histories between the 2 groups receiving SGLT2 inhibitors or DPP4 inhibitors. Comorbidities included hypertension, hyperlipidemia, cerebral vascular disease, coronary artery disease, obesity, and chronic kidney disease (CKD), which had occurred over at least 3 ambulatory care visits or 1 inpatient care visit within 1 year of the index date. Apart from use of SGLT2 inhibitors or DPP4 inhibitors, other medicines, such as diuretics, glucagonlike peptide-1 (GLP-1) agonists, insulin, metformin, and statins, that were used before the index year were adjusted in our models. All patients were followed up from the index date until gout diagnosis, death, or study end (ie, December 31, 2018), whichever occurred first. Data analysis was conducted from April 1 to June 30, 2021.

### Statistical Analysis

Descriptive data, including mean (SD) for continuous variables and numbers and frequencies for categorical variables, were calculated for the groups. Propensity scores for the maximum likelihood estimations with SGLT2 inhibitor treatment were determined by multiple logistic regression analysis, conditional on baseline covariates. One-to-one greedy matching was implemented with caliper width equal to 0.2.^13^ Standardized mean differences (SMDs) with a cutoff value of 0.10 were then adopted to assess the balance of covariates between 2 groups in the overall population and in the propensity score–matched population.^14^ Survival curves of gout incidence in SGLT2 inhibitor and DPP4 inhibitor users were plotted in the overall study population and the propensity score–matched population using the Kaplan-Meier method. Also, the difference between the curves was compared by log-rank test. Crude and adjusted hazard ratios (HRs) and 95% CIs for gout risk were evaluated using univariate and multiple Cox proportional hazards regression models. Related confounders, such as age, sex, hypertension, hyperlipidemia, cerebral vascular disease, coronary artery disease, obesity, CKD, diuretics, and use of GLP-1 agonists, insulin, metformin, and statins, were adjusted in multiple regression models. We further explored different gout risk stratifications by various SGLT2 inhibitor types (dapagliflozin, empagliflozin, and canagliflozin). In addition, post hoc subgroup analyses by baseline comorbidities and other medication use were conducted using multiple Cox proportional hazards regression models. Multiplicative interactions between SGLT2 inhibitor use and baseline comorbidities and other medications associated with gout risk were calculated by adding cross-product terms in multiple Cox proportional hazards regression models. We then performed sensitivity analyses to define gout incidence using a different gout definition. A gout diagnosis was determined using a diagnostic code in addition to prescribed gout-specific medicines or acute medicines, including nonsteroidal anti-inflammatory drugs and corticosteroids, to treat the condition up to 14 days after diagnosis. All statistical analyses were conducted using SAS, version 9.4 statistical software (SAS Institute Inc). Statistical significance was defined as a 2-sided *P* < .05 value.

## Results

**Figure 2.**
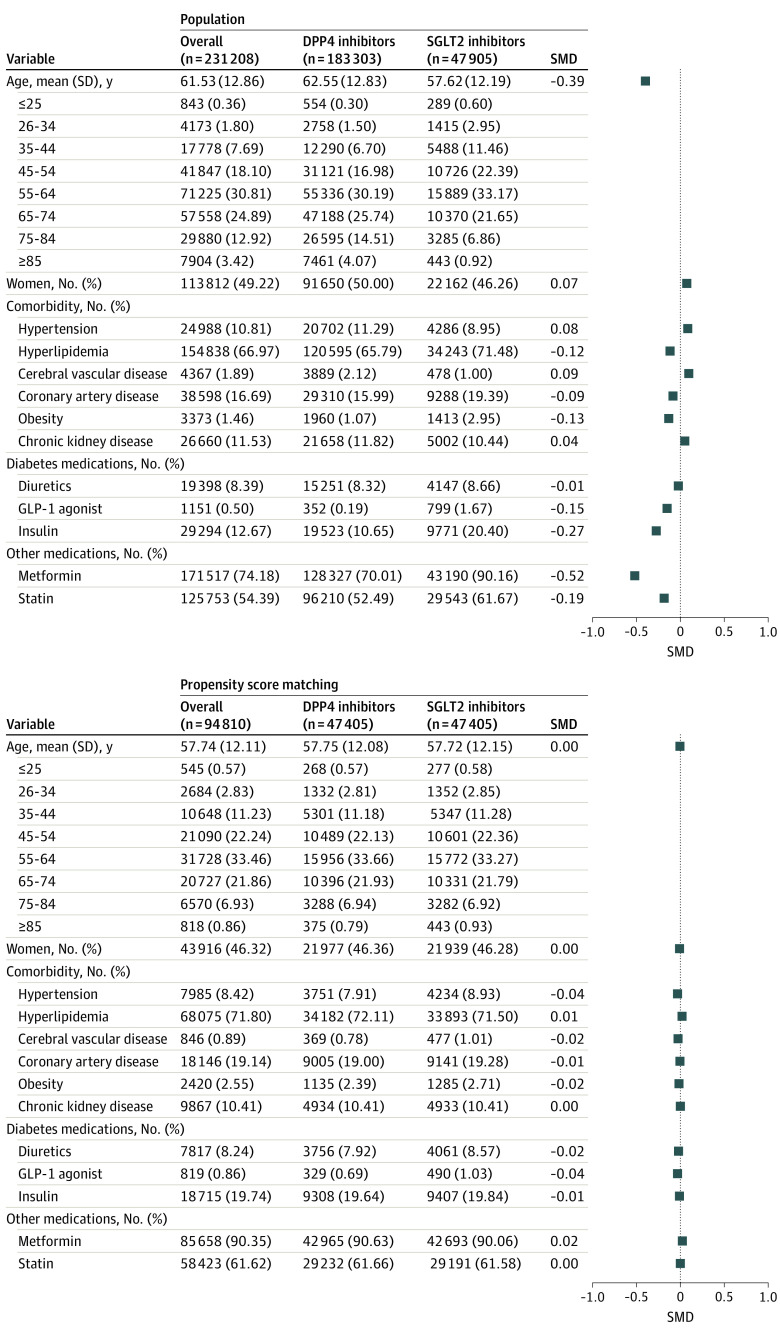
Demographic Profiles of Patients With Type 2 Diabetes Using Dipeptidyl Peptidase 4 (DPP4) Inhibitors and Sodium-Glucose Cotransporter 2 (SGLT2) Inhibitors in the Overall Study Population and the Propensity Score–Matched Population GLP-1 indicates glucagonlike peptide-1; SMD, standardized mean difference.

A total of 231 208 patients with T2DM, including 47 905 patients (20.72%) using SGLT2 inhibitors and 183 303 patients (79.28%) using DPP4 inhibitors were identified in the overall population. The overall mean (SD) age was 61.53 (12.86) years; the SGLT2 inhibitor group was younger (mean [SD], 57.62 [12.19] vs 62.55 [12.83] years). A total of 113 812 patients (49.22%) were women; the SGLT2 inhibitor group contained fewer women compared with the DPP4 inhibitor group (22 162 [46.26%] vs 91 650 [50.00%]) (Figure 2). Patients receiving SGLT2 inhibitors were more likely to have high levels of hyperlipidemia and obesity and to be simultaneously receiving GLP-1 agonists, insulin, metformin, and statins compared with those receiving DPP4 inhibitors.

To increase comparability between groups, we constructed 47 405 pairs of individuals receiving an SGLT2 inhibitor or a DPP4 inhibitor using propensity score–matched analyses. For this population, the mean (SD) age was 57.74 (12.11) years and 43 916 (46.32%) were women. In addition, the balances of comorbidities and medicine use between SGLT2 inhibitor and DPP4 inhibitor users were assessed by SMDs. An SMD value below 0.10 was considered a negligible difference between the 2 groups.

### SGLT2 Inhibitor Use and Gout Risk

**Figure 3.**
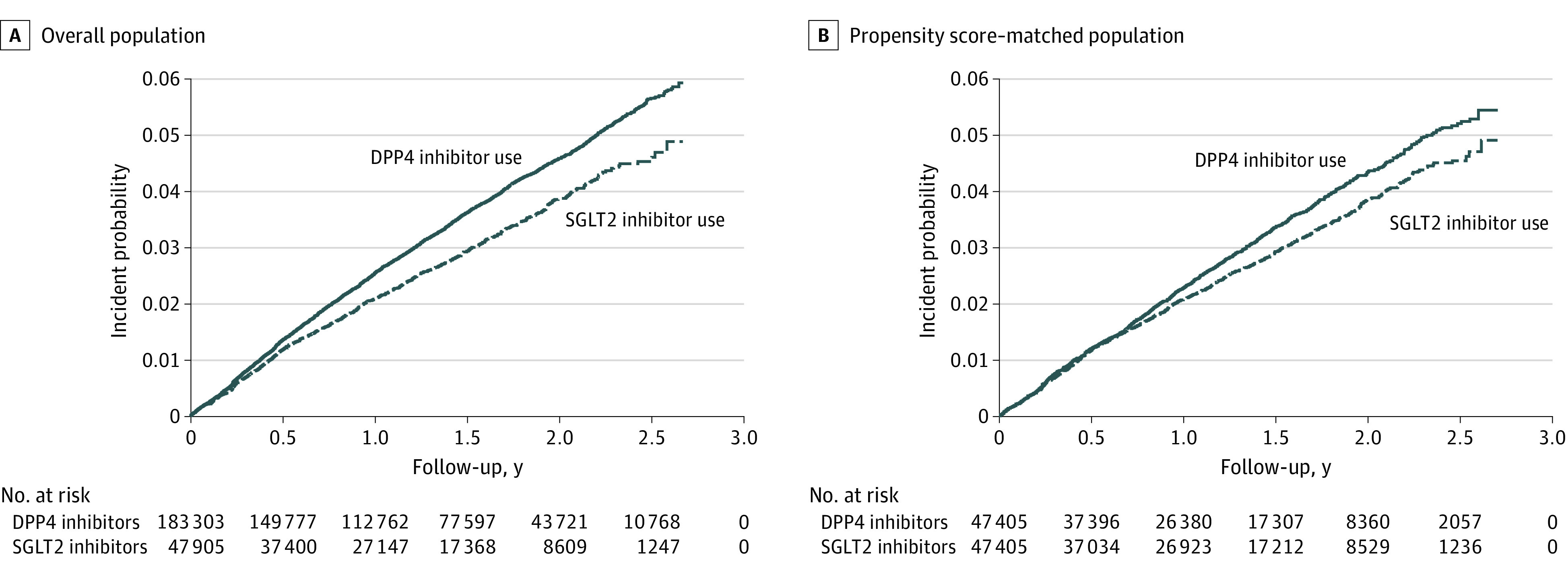
Gout Incidence in Patients With Type 2 Diabetes Receiving Dipeptidyl Peptidase 4 (DPP4) Inhibitors and Sodium-Glucose Cotransporter 2 (SGLT2) Inhibitors Gout incidence in the overall study population (A) and the propensity score–matched population (B).

During a follow-up of approximately 2.5 years, we observed differences in the overall gout incidence: 20.26 per 1000 patient-years for SGLT2 inhibitor users and 24.30 per 1000 patient-years for DPP4 inhibitor users. The cumulative incidence of gout between the propensity score–matched populations was 4.63% for SGLT2 inhibitors and 5.25% for DPP4 inhibitors (log-rank *P* = .005) (Figure 3). Similar differences were also seen in the overall population (SGLT2 inhibitor, 4.61%; DPP4 inhibitor, 5.67%; log-rank *P* < .001).

In the propensity score–matched population, we observed a 0.89-fold risk of gout in patients receiving SGLT2 inhibitors compared with DPP4 inhibitors using both univariate (HR, 0.89; 95% CI, 0.82-0.97; *P* = .005) and multiple Cox proportional hazards regression (HR, 0.89; 95% CI, 0.82-0.96; *P* = .004) models (Table). Similar associations between SGLT2 inhibitor use and a lower gout risk were also indicated in the overall study population (HR, 0.87; 95% CI, 0.81-0.93; *P* < .001). For SGLT2 inhibitor prescriptions, 55.9% were for dapagliflozin, followed by empagliflozin (43.0%) and canagliflozin (1.1%). After considering potential risk factors, dapagliflozin users had an HR of 0.86 (95% CI, 0.78-0.95, *P* = .002), and empagliflozin users had an HR of 0.93 (95% CI, 0.84-1.03, *P* = .16) for incidence of gout compared with DPP4 inhibitor users. No associations between canagliflozin use and gout risk were observed regardless of the propensity score–matched population or the overall study population.

**Table.** Incidence and Risk of Gout Between Patients With DPP4 and With SGLT2 Inhibitor Use in Patients With Type 2 Diabetes^a^

Variable	No.	Events	Person-years	Incident rate^b^	Crude HR (95% CI)	*P* value	Adjusted HR (95% CI)^c^	*P* value
**All study population without matching**								
Outcome: gout								
DPP4 inhibitor	183 303	5874	241 736.97	24.30	1 [Reference]		1 [Reference]	
SGLT2 inhibitor	47 905	1165	57 494.91	20.26	0.83 (0.78-0.88)	<.001	0.87 (0.81-0.93)	<.001
Dapagliflozin	26 778	626	32 516.44	19.25	0.79 (0.73-0.86)	<.001	0.84 (0.77-0.92)	<.001
Empagliflozin	20 598	537	24 820.52	21.64	0.88 (0.81-0.97)	.006	0.90 (0.83-0.99)	.03
Canagliflozin	529	2	157.98	12.66	0.48 (0.12-1.91)	.30	0.51 (0.13-2.02)	.34
Outcome: gout and medicine (in 14 d)								
DPP4 inhibitor	183 303	1902	245 378.32	7.75	1 [Reference]		1 [Reference]	
SGLT2 inhibitor	47 905	395	58 147.3	6.79	0.87 (0.78-0.97)	.01	0.85 (0.76-0.95)	.004
Dapagliflozin	26 778	226	32 853.96	6.88	0.88 (0.77-1.01)	.07	0.88 (0.76-1.01)	.07
Empagliflozin	20 598	169	25 134.98	6.72	0.86 (0.73-1.00)	.06	0.82 (0.70-0.96)	.02
Canagliflozin	529	0	158.35	NA	NA	NA	NA	NA
**Study population with propensity score matching**								
Outcome: gout								
DPP4 inhibitor	47 405	1307	57 394.84	22.77	1 [Reference]		1 [Reference]	
SGLT2 inhibitor	47 405	1158	56 947.38	20.33	0.89 (0.82-0.97)	.005	0.89 (0.82-0.96)	.004
Dapagliflozin	26 511	620	32 200.18	19.25	0.85 (0.77-0.93)	<.001	0.86 (0.78-0.95)	.002
Empagliflozin	20 371	537	24 590.93	21.84	0.96 (0.87-1.06)	.40	0.93 (0.84-1.03)	.16
Canagliflozin	523	1	156.27	6.40	0.26 (0.04-1.83)	.18	0.26 (0.04-1.86)	.18
Outcome: gout and medicine (in 14 d)								
DPP4 inhibitor	47 405	466	58 142.09	8.01	1 [Reference]		1 [Reference]	
SGLT2 inhibitor	47 405	393	57 593.52	6.82	0.85 (0.74-0.97)	.02	0.85 (0.74-0.97)	.02
Dapagliflozin	26 511	224	32 531.75	6.89	0.86 (0.73-1.01)	.06	0.88 (0.75-1.03)	.10
Empagliflozin	20 371	169	24 905.50	6.79	0.85 (0.71-1.01)	.06	0.83 (0.69-0.98)	.03
Canagliflozin	523	0	156.27	NA	NA	NA	NA	NA

Abbreviations: DPP4, dipeptidyl peptidase 4; HR, hazard ratio; NA, not applicable; SGLT2, sodium-glucose cotransporter 2.

^a^ Analyses were conducted using Cox proportional hazards regression models.

^b^ Incident rates were calculated as events of gout per 100 person-years.

^c^ Models were adjusted for age, sex, hypertension, hyperlipidemia, cerebral vascular disease, coronary artery disease, obesity, chronic kidney disease, and use of diuretics, glucagonlike peptide-1 agonists, insulin, metformin, and statins.

**Figure 4.**
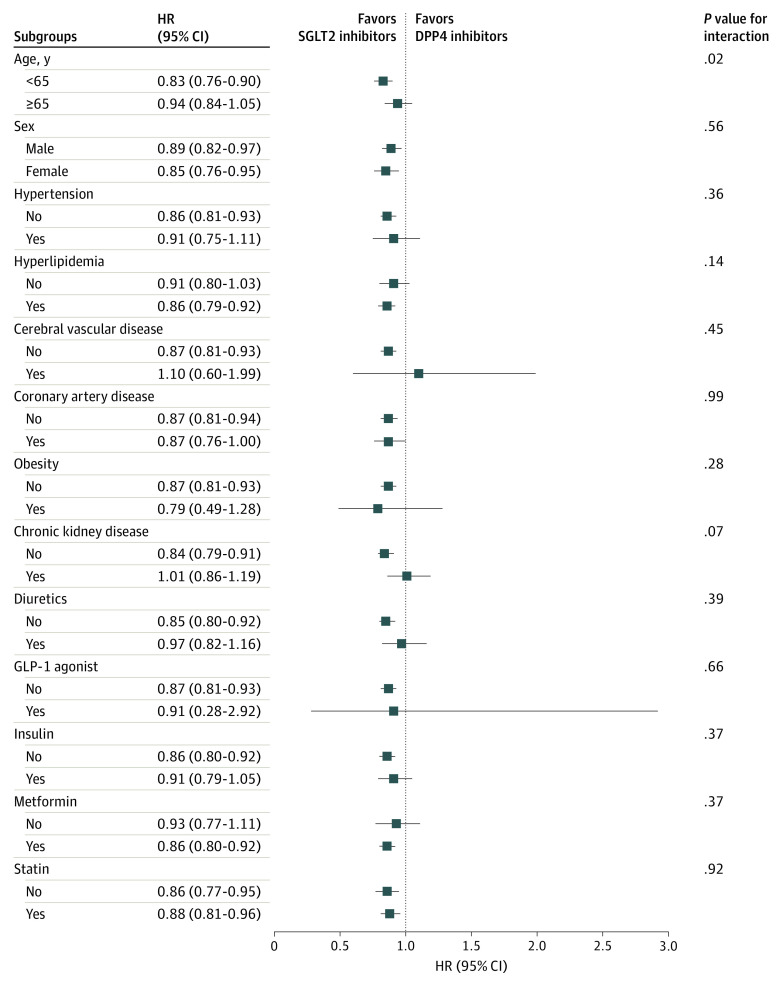
Interaction Analysis of Gout Incidence in Patients With Type 2 Diabetes Receiving Dipeptidyl Peptidase 4 (DPP4) Inhibitors and Sodium-Glucose Cotransporter 2 (SGLT2) Inhibitors Using Baseline Comorbidities and Other Medications in the Propensity Score–Matched Population GLP-1 indicates glucagonlike peptide-1; HR, hazard ratio.

We performed interaction analyses of various comorbidities and other medicine use with SGLT2 inhibitors for evaluation of gout risk in the propensity score–matched population (Figure 4). First, for subgroup analysis of individuals younger than 65 years, SGLT2 inhibitor users had a significantly lower risk of gout (HR, 0.83; 95% CI, 0.76-0.90), but there was no association between SGLT2 inhibitor use and gout for those aged 65 years or older. Furthermore, interactions were evident between SGLT2 inhibitor use and the younger age group in terms of gout risk. This finding suggests that the association between SGLT2 inhibitor use and gout was affected by age (<65 or ≥65 years). However, other comorbidities and medicine use did not statistically interact with SGLT2 inhibitor use for gout risk (all *P* > .05). These results suggest that SGLT2 inhibitor benefits in patients with T2DM for a lower risk of gout were not different across subgroups.

### Sensitivity Analyses

In sensitivity analyses, gout outcomes were defined in patients receiving gout-specific medicine or acute medicine up to 14 days after gout diagnosis; these analyses were conducted to test the robustness of our findings. Propensity score–matched population results indicated SGLT2 inhibitors were associated with a lower risk of gout by 15%. We observed a significant 0.85-fold (95% CI, 0.74-0.97; *P* = .02) risk for gout in patients receiving SGLT2 inhibitors compared with DPP4 inhibitors (Table), especially for those receiving empagliflozin (HR, 0.83; 95% CI, 0.69-0.98; *P* = .03).

## Discussion

The findings of this study showed that use of SGLT2 inhibitors in T2DM was associated with a lower risk of gout incidence compared with use of DPP4 inhibitors in a national Taiwanese cohort. After adjustment for potential confounders, gout incidence was reduced by 11% with SGLT2 inhibitors. A sensitivity analysis, in which a gout diagnosis was conducted using an *ICD-9-CM* or *ICD-10-CM* code with gout-related medication, showed a lower risk of gout incidence by 15% for SGLT2 inhibitor use.

Several factors likely accounted for the association between a lower gout risk during SGLT2 inhibitor administration. First, SGLT2 inhibitors consistently lowered blood urate levels in large randomized clinical trials.^15,16,17^ Hyperuricemia appears to be the main risk factor for gout; therefore, baseline hyperuricemia^18^ or posttreatment blood urate levels^19^ have been shown to predict gout occurrence. Previous studies indicated that SGLT2 inhibitors enhanced urine urate secretion in tubular fluid, possibly by inhibiting kidney tubular urate transporters, such as URAT1, with increased luminal glucose delivery also promoting urine urate secretion.^20^ Next, SGLT2 inhibitors are likely to enhance sirtuin-1,^21^ which inhibits xanthine oxidase and decreases blood urate levels.^22^ In addition, acute gout flares activate the NOD-, LRR-, and pyrin domain-containing 3 (NLRP3) inflammasome and promotes interleukin-1β.^23^ The SGLT2 inhibitors significantly suppressed NLRP3 inflammasome activation and subsequent IL-1β secretion in patients with T2DM.^24^

To our knowledge, one US study^25^ has noted that the gout incidence risk rate was reduced by more than 30% in patients receiving SGLT2 inhibitors compared with prescribed GLP-1 agonists; this risk reduction rate was larger than the rate shown with our data. Several possible explanations exist for this difference. First, all GLP-1 agonists do not affect blood urate levels.^26^ In contrast, studies reported some DPP4 inhibitors may inhibit blood urate levels.^27,28^ As a positive control in our study, DPP4 inhibitors may mitigate the SGLT2 inhibitor association with gout reduction. A meta-analysis^29^ reported that blood urate reductions were rapid after 4 weeks and were persistent with long-term SGLT2 inhibitor treatment after 2 years. This outcome was also observed in our study, and lower gout incidence association with SGLT2 inhibitors was persistent. This meta-analysis also reported that any type of SGLT2 inhibitor significantly decreased blood urate levels compared with controls. In our study, empagliflozin and dapagliflozin were associated with a lower risk of gout incidence. Canagliflozin also appeared to exhibit a protective outcome, but this finding was not statistically significant.

Subgroup baseline characteristics analyses noted that an association between SGLT2 inhibitors and a lower risk of gout was present in significantly more younger (age <65 years) than older (age ≥65 years) age groups (*P* = .01 for interaction). A previous study showed use of SGLT2 inhibitors tended to decrease higher blood urate levels in younger than older age groups.^29^ Studies reported that blood urate–lowering effects were not apparent in patients with CKD,^30,31^ possibly explaining no associations between SGLT2 inhibitors and gout incidence (HR, 1.01; 95% CI, 0.86-1.19) in patients with CKD in our study. Although patients without other comorbidities tended to take greater advantage of receiving SGLT2 inhibitors and lower gout incidence, our results noted that the benefit of SGLT2 inhibitors in patients with T2DM for lower risk of gout were not significantly different across subgroups.

### Strengths and Limitations

Our study strengths included a large sample size, nationwide scope, and over 99% coverage of 23 million Taiwanese individuals. In Taiwan, diagnostic accuracy of diabetes in health insurance claims data has been validated.^9^ The validity of gout diagnoses using *ICD-9-CM* and *ICD-10-CM* codes was also reported in previous research.^11^ In addition, patients with previous gout diagnoses and those using gout-related medications were excluded to avoid any confounding with determination of outcomes.

Our study also had limitations. First, detailed laboratory values (eg, blood urate levels) are not included in the National Health Insurance database, potentially influencing study outcomes. Second, in Taiwan, SGLT2 inhibitors were introduced in 2016; therefore, the sample size and event rate were decreased in the subgroup analysis, which would have lessened the power to detect differences. Third, observation and outcome periods were relatively short. However, a significant association of a lower risk of gout with SGLT2 inhibitors was still observed. Further studies with longer follow-up durations should be implemented. We would expect this incidence difference to grow with longer follow-up periods because reductions in blood urate levels should translate to lower incident gout risks over time. Fourth, gout *ICD-9-CM* and *ICD-10-CM* codes were not validated in our database. In addition, a previous validation study was limited by the uncertainty of external validity criteria in the general population.^11^ Nevertheless, a gout diagnosis was strengthened by use of *ICD-9-CM* and *ICD-10-CM* codes in combination with medication information, which also noted an association between SGLT2 inhibitor and a lower risk of gout.

## Conclusions

The findings of this study suggest that use of SGLT2 inhibitors in patients with T2DM is associated with a lowered gout incidence of 11% compared with DPP4 inhibitors. The SGLT2 inhibitors appeared to protect patients with T2DM patients from gout, even across subgroups.

## Notes

Supplement. **eTable.** Covariates Used for Cohort Creation and Study Outcome
