# Supplementary material for: Association of Sodium-Glucose Transport Protein 2 Inhibitor Use for Type 2 Diabetes and Incidence of Gout in Taiwan
Source: JAMA Netw Open. 2021 Nov 19;4(11):e2135353. doi: 10.1001/jamanetworkopen.2021.35353 (PMC8605485; doi:10.1001/jamanetworkopen.2021.35353)
Supplement: Supplement. — eTable. Covariates Used for Cohort Creation and Study Outcome [file jamanetwopen-e2135353-s001.pdf]

Supplementary Online Content

Chung MC, Hung PH, Hsiao PJ, et al. Association of sodium-glucose transport protein 2 inhibitor use for type 2 diabetes and incidence of gout in Taiwan. *JAMA Netw Open*. 2021;4(11):e2135353.
doi:10.1001/jamanetworkopen.2021.35353

eTable. Covariates Used for Cohort Creation and Study Outcome

This supplementary material has been provided by the authors to give readers additional information about their work.

© 2021 Chung MC et al. *JAMA Network Open.*

eTable. Covariates used for cohort creation and study outcome.

Type 2 Diabetes Mellitus	ICD-09:<br>"250.0","250.00","250.02","250.10","250.12","250.2","250.20","250.22","250.30","250.32","250.40","250.42","250.50","250.52","250.60","250.62","250.70","250.72","250.80","250.82","250.90","250.92".<br><br>ICD-10:<br>"E11","E110","E110.0","E110.1","E112","E112.1","E112.2","E112.9","E113","E113.1","E113.11","E113.19","E113.2","E113.21","E113.29","E113.3","E113.31","E113.39","E113.4","E113.41","E113.49","E113.5","E113.51","E113.59","E113.6","E113.9","E114","E114.0","E114.1","E114.2","E114.3","E114.4","E114.9","E115","E115.1","E115.2","E115.9","E116","E116.1","E116.10","E116.18","E116.2","E116.20","E116.21","E116.22","E116.28","E116.3","E116.30","E116.38","E116.4","E116.41","E116.49","E116.5","E118","E119".
Diagnosis of gout	ICD-09:<br>"274.00","274.01","274.02","274.03","274.81","274.82","274.89","274.9".<br><br>ICD-10:<br>"M10.00","M10.9","M10.0","M10.01","M10.011","M10.012","M10.019","M10.02","M10.021","M10.022","M10.029","M10.03","M10.031","M10.032","M10.039","M10.04","M10.041","M10.042","M10.049","M10.05","M10.051","M10.052","M10.059","M10.06","M10.061","M10.062","M10.069","M10.07","M10.071","M10.072","M10.079","M10.08","M10.09","M10.2","M10.20","M10.21","M10.211","M10.212","M10.219","M10.22","M10.221","M10.222","M10.229","M10.23","M10.231","M10.232","M10.239","M10.24","M10.241","M10.242","M10.249","M10.25","M10.251","M10.252","M10.259","M10.26","M10.261","M10.262","M10.269","M10.27","M10.271","M10.272","M10.279","M10.28","M10.29","M10.4","M10.40","M10.41","M10.411","M10.412","M10.419","M10.42","M10.421","M10.422","M10.429","M10.43","M10.431","M10.432","M10.439","M10.44","M10.441","M10.442","M10.449","M10.45","M10.451","M10.452","M10.459","

© 2021 Chung MC et al. *JAMA Network Open.*

	M10.46","M10.461","M10.462","M10.469","M10.47","M10.471","M10.472","M10.479","M10.48","M10.49","M1A.0","M1A.00","M1A.00X0","M1A.00X1","M1A.01","M1A.011","M1A.0110","M1A.0111","M1A.012","M1A.0120","M1A.0121","M1A.019","M1A.0190","M1A.0191","M1A.02","M1A.021","M1A.0210","M1A.0211","M1A.022","M1A.0220","M1A.0221","M1A.029","M1A.0290","M1A.0291","M1A.03","M1A.031","M1A.0310","M1A.0311","M1A.032","M1A.0320","M1A.0321","M1A.039","M1A.0390","M1A.0391","M1A.04","M1A.041","M1A.0410","M1A.0411","M1A.042","M1A.0420","M1A.0421","M1A.049","M1A.0490","M1A.0491","M1A.05","M1A.051","M1A.0510","M1A.0511","M1A.052","M1A.0520","M1A.0521","M1A.059","M1A.0590","M1A.0591","M1A.06","M1A.061","M1A.0610","M1A.0611","M1A.062","M1A.0620","M1A.0621","M1A.069","M1A.0690","M1A.0691","M1A.07","M1A.071","M1A.0710","M1A.0711","M1A.072","M1A.0720","M1A.0721","M1A.079","M1A.0790","M1A.0791","M1A.08","M1A.08X0","M1A.08X1","M1A.09","M1A.09X0","M1A.09X1","M1A.2","M1A.20","M1A.20X0","M1A.20X1","M1A.21","M1A.211","M1A.2110","M1A.2111","M1A.212","M1A.2120","M1A.2121","M1A.219","M1A.2190","M1A.2191","M1A.22","M1A.221","M1A.2210","M1A.2211","M1A.222","M1A.2220","M1A.2221","M1A.229","M1A.2290","M1A.2291","M1A.23","M1A.231","M1A.2310","M1A.2311","M1A.232","M1A.2320","M1A.2321","M1A.239","M1A.2390","M1A.2391","M1A.24","M1A.241","M1A.2410","M1A.2411","M1A.242","M1A.2420","M1A.2421","M1A.249","M1A.2490","M1A.2491","M1A.25","M1A.251","M1A.2510","M1A.2511","M1A.252","M1A.2520","M1A.2521","M1A.259","M1A.2590","M1A.2591","M1A.26","M1A.261","M1A.2610","M1A.2611","M1A.262","M1A.2620","M1A.2621","M1A.269","M1A.2690","M1A.2691","M1A.27","M1A.271","M1A.2710","M1A.2711","M1A.272","M1A.2720","M1A.2721","M1A.279","M1A.2790","M1A.2791","M1A.28","M1A.28X0","M1A.28X1","M1A.29","M1A.29X0","M1A.29X1","M1A.4","M1A.40","M1A.40X0","M1A.40X1","M1A.41","M1A.411","M1A.4110","M1A.4111","M1A.412","M1A.4120","M1A.4121","M1A.419","M1

© 2021 Chung MC et al. *JAMA Network Open.*

	A.4190","M1A.4191","M1A.42","M1A.421","M1A.4210"," M1A.4211","M1A.422","M1A.4220","M1A.4221","M1A.429","M1A.4290","M1A.4291","M1A.43","M1A.431","M1A.4310","M1A.4311","M1A.432","M1A.4320","M1A.4321","M1A.439","M1A.4390","M1A.4391","M1A.44","M1A.441"," M1A.4410","M1A.4411","M1A.442","M1A.4420","M1A.4421","M1A.449","M1A.4490","M1A.4491","M1A.45","M1A.451","M1A.4510","M1A.4511","M1A.452","M1A.4520","M1A.4521","M1A.459","M1A.4590","M1A.4591","M1A.46"," M1A.461","M1A.4610","M1A.4611","M1A.462","M1A.4620","M1A.4621","M1A.469","M1A.4690","M1A.4691","M1A.47","M1A.471","M1A.4710","M1A.4711","M1A.472","M1A.4720","M1A.4721","M1A.479","M1A.4790","M1A.4791","M1A.48","M1A.48X0","M1A.48X1","M1A.49","M1A.49X0","M1A.49X1","M1A.9","M1A.9XX0","M1A.9XX1".

© 2021 Chung MC et al. *JAMA Network Open.*
